# Influence of aerobic exercise training on post-exercise responses of aortic pulse pressure and augmentation pressure in postmenopausal women

**DOI:** 10.3389/fphys.2015.00268

**Published:** 2015-10-07

**Authors:** Nobuhiko Akazawa, Song-Gyu Ra, Jun Sugawara, Seiji Maeda

**Affiliations:** ^1^Faculty of Health and Sport Sciences, University of TsukubaTsukuba, Japan; ^2^Japanese Society for the Promotion of ScienceTokyo, Japan; ^3^Human Informatics Research Institute, National Institute of Advanced Industrial Science and TechnologyTsukuba, Japan

**Keywords:** augmentation pressure, aortic blood pressure, aerobic exercise, aerobic training, postmenopausal women

## Abstract

Central arterial blood pressure (BP) is more predictive of future cardiovascular events than is brachial BP because it reflects the BP load imposed on the left ventricle with greater accuracy. However, little is known about the effects of exercise training on central hemodynamic response to acute exercise. The purpose of the present study was to determine the influence of an aerobic exercise regimen on the response of aortic BP after a single aerobic exercise in postmenopausal women. Nine healthy postmenopausal women (age: 61 ± 2 years) participated in a 12-week aerobic exercise training regimen. Before and after the training, each subjects performed a single bout of cycling at ventilatory thresholds for 30 min. We evaluated the post-exercise aortic BP response, which was estimated via the general transfer function from applanation tonometry. After the initial pre-training aerobic exercise session, aortic BP did not change significantly: however, aortic pulse pressure and augmentation pressure were significantly attenuated after the single aerobic exercise session following the 12-week training regimen. The present study demonstrated that a regular aerobic exercise training regimen induced the post-exercise reduction of aortic pulse pressure and augmentation pressure. Regular aerobic exercise training may enhance post-exercise reduction in aortic BP.

## Introduction

Aging can increase both blood pressure (BP) and arterial stiffness, both of which are major risk factors for cardiovascular disease (Blacher et al., 1999; O'Rourke, 1999). Central BP, including aortic or carotid pressures, reportedly has a greater influence on cardiovascular disease than dose peripheral BP (Roman et al., 2007). In addition, the pulsatile component of central arterial pressure (e.g., pulse pressure) is strongly correlated with cardiovascular events and outcome than is systolic pressure (Benetos et al., 1998; Safar et al., 2002; Roman et al., 2007). Central aortic pulse pressure can be subdivided into amplitude of the first systolic peak and augmentation pressure. The augmentation pressure is influenced by aging, body height, and arterial stiffness (Nichols and Edwards, 2001; Nichols and O'Rourke, 2005). In women, the increase in arterial stiffness with age accelerates around menopause (Tomiyama et al., 2003). In addition, the increase in central pulse pressure in women is much greater than that in men (McEniery et al., 2005). Therefore, the management of central BP, especially pulse pressure and argumentation pressure, may be of great pathophysiological importance in postmenopausal women.

Exercise is beneficial for the treatment of BP and vascular aging. A single bout of aerobic exercise can cause a transient reduction in peripheral BP (Kaufman et al., 1987; MacDonald, 2002). Kingwell et al. (1997) reported that arterial stiffness decreases after acute aerobic exercise in young men. Recently, we demonstrated that a single bout of aerobic exercise decreased not only arterial stiffness but also the central pulse pressure in young men (Sugawara et al., 2015). Alternatively, a previous study by our group reported that arterial stiffness in older women did not change after acute aerobic exercise: however, after a period of regular exercise training, arterial stiffness was significantly decreased after acute aerobic exercise (Maeda et al., 2008). This study implied that long-term exercise training may enhance the cardiovascular response to acute exercise. However, little is known about the effect of exercise training on the post-exercise response of central hemodynamics in older women.

The present study aimed to determine the effect of exercise training on the central BP response to an acute single bout of aerobic cycling. We hypothesize that persistent exercise training can enhance the aortic BP response to a single bout of aerobic exercise. To test our hypothesis, we measured aortic pulse pressure and augmentation pressure during recovery from a single bout of exercise before and after 12 weeks of aerobic exercise training.

## Methods

### Subjects

Nine sedentary postmenopausal women (52-66 years old) participated in the study. The subjects were nonsmokers, non-obese, and free of cardiovascular disease, as assessed by medical history. None of the subjects were taking medications affecting the cardiovascular system or hormone replacement therapy. All potential risks associated with the study were explained to the subjects, and they gave written informed consent for participation in the study. All procedures were reviewed and approved by the ethical committee of the University of Tsukuba.

### Experimental protocol

All 9 subjects completed an aerobic exercise training regimen. Before and after aerobic exercise training, each subject performed an acute exercise test that consisted of a 30-min aerobic cycling exercise at the intensity of the individual's ventilatory threshould (VT). All participants were at least 3 h postprandial, and did not consume caffeine and alcohol for 12-h and strenuous exercise for 24 h. We measured brachial BP, central BP and heart rate (HR) before and 30 and 60 min after the single bout of exercise. At least 2 days before the acute exercise test, VT and blood chemistries were measured after overnight fast. All measurements were performed at a constant room temperature (24-26°C).

### Measurement

Pressure waveforms were obtained simultaneously in the common carotid artery using an applanation tonometry sensor (FormPWV/ABI, Colin Medical Technology, Komaki, Japan). Carotid arterial pressure waveforms were sampled at 1000 Hz for off line analysis and resampled at 128 Hz with data analysis software (AcqKnowledge, BIOPAC system Santa Barbara, CA) (Sugawara et al., 2010). And then, pressure waveform transferred into aortic pressure waveforms with an arterial waveform analysis software involving a validated generalized transfer function (SphygmoCor software, AtCor Medical, Sydney, Australia) (Karamanoglu et al., 1993). Pressure waveforms were calibrated to brachial mean arterial pressure and diastolic BP. To qualify the magnitude of wave reflection from the periphery to the heart, the first and second systolic peak, defined as P1 and P2, respectively, of the aortic pressure waveforms were analyzed. Aortic systolic arterial pressure, diastolic arterial pressure, and augmentation pressure (peak pressure—pressure at the inflection point at systolic shoulder) was computed from synthesized aortic pressure waveforms. Pulse pressure was calculated systolic blood pressure—diastolic blood pressure. The day-to-day coefficient of variation for systolic blood pressure, diastolic blood pressure, pulse pressure and augmentation pressure was 4 ± 1, 5 ± 1, 6 ± 2, and 11 ± 3%, respectively.

Carotid-femoral pulse wave velocity (cfPWV) was measured as arterial stiffness by a semi-automated vascular testing system (Sugawara et al., 2015). Briefly, carotid and femoral pressure waveforms were obtained by two applanation tonometry sensors incorporating an array of 15 transducers (Form PWV/ABI, Colin Medical Technology, Komaki, Japan). The distance between the left common carotid and left common femoral arterial recording sites divided by the transit time resulted in the calculation of cfPWV.

VT was measured during the incremental cycle ergometer exercise by using online computer-assisted circuit spirometry (AE300S; Minato Medical Science, Osaka, Japan). All subjects performed a symptom-limited cycling exercise test (after a 2 min warm-up at 20 W, followed by 10 W increases every min) until they felt exhaustion or reached their age predicted maximal HR. The peak oxygen consumption (VO_2peak_) was defined at the highest value recorded during the test. Each individual VT was calculated by using regression analysis of the slopes of carbon dioxide production, oxygen uptake and the minute ventilation plot.

A blood sample was collected from the antecubital vein after overnight fasting. Serum total cholesterol, triglycerides, and plasma glucose were determined using standard enzymatic technique.

### Aerobic exercise training regimen

The subjects underwent aerobic exercise training 4-6 days per week (three supervised sessions and additional home-based sessions) for a total 12 weeks (Akazawa et al., 2012). Initially, the subjects performed cycling and walking sessions for 30 min/day at a relatively low intensity (60% of their individually determined maximal HR). As their exercise tolerance improved, the intensity and duration of aerobic exercise was increased to 40–60 min/day at an intensity of 70-80% of the maximal HR.

### Steady-state aerobic exercise test

Before and after the exercise training program, the subjects performed a steady-state exercise test at their individual VT for 30 min using an electrically braked cycle ergometer (75XLIII; Combi Welness, Tokyo, Japan). An investigator monitored the subject's working load, oxygen uptake, heart rare, and rating of perceived exertion (RPE) during exercise and supervised each subject to perform the cycling exercise around the target intensities at 60 rpm.

### Statistical analyses

Data are expressed as mean ± SE. Student's *t*-test for paired data was used to evaluate the difference in the body mass, blood chemistry, VT, and VO_2peak_ at test and oxygen uptake, heart rate, and RPE during a single bout of exercise before and after exercise training. A two-way analysis of variance with repeated measures was performed to identify an interaction or main effect. A Dunnet post hoc test was used, when indicated for a significant main effect or interaction. Univariable correlation analyses were used to determine the relations between variables of interest. Statistical significance was set a priori at *P* < 0.05 for all comparisons.

## Results

There were no significant differences in measurement of body mass, triglyceride and blood glucose between before and after the 12-week exercise training regimen (Table 1). However, after the regimen, total cholesterol was significantly decreased and individual VT and VO_2peak_ were significantly increased (Table 1).

**Table 1:** **Subjects characteristics before and after exercise training**.

	**Before training**	**After training**
Age (years)	61±2	
Height (cm)	154±1	
Weight (kg)	52±2	51±1
Total cholesterol (mg/dL)	231±9	216±7^*^
Triglyceride (mg/dL)	127±42	138±28
Blood glucose (mg/dL)	94±2	92±2
Oxygen uptake at VT (mL/kg/min)	12±2	16±2^*^
Peak oxygen uptake (mL/kg/min)	23±1	26±1^*^

*Data are means ± SE. VT, ventilatory threshold*.

* *P < 0.05 vs. before training*.

The oxygen uptake and heart rate increased during exercise and increase in oxygen uptake and heart rate was higher after exercise training than that of before training, but RPE was not different from between before and after training regimen (Table 2).

**Table 2:** **Work load, oxygen uptake, heart rate, and rating of perceived exertion during 30 min steady-state exercise**.

	**Before training**	**After training**
Work load (Watt)	31±4	59±3^*^
Oxygen uptake (mL/kg/min)	12±1	17±1^*^
Heart rate (bpm)	106±5	125±4^*^
Rating of perceived exertion	13±1	13±1

*Data are means ± SE*.

* *P < 0.05 vs. before training*.

**Figure 1:**
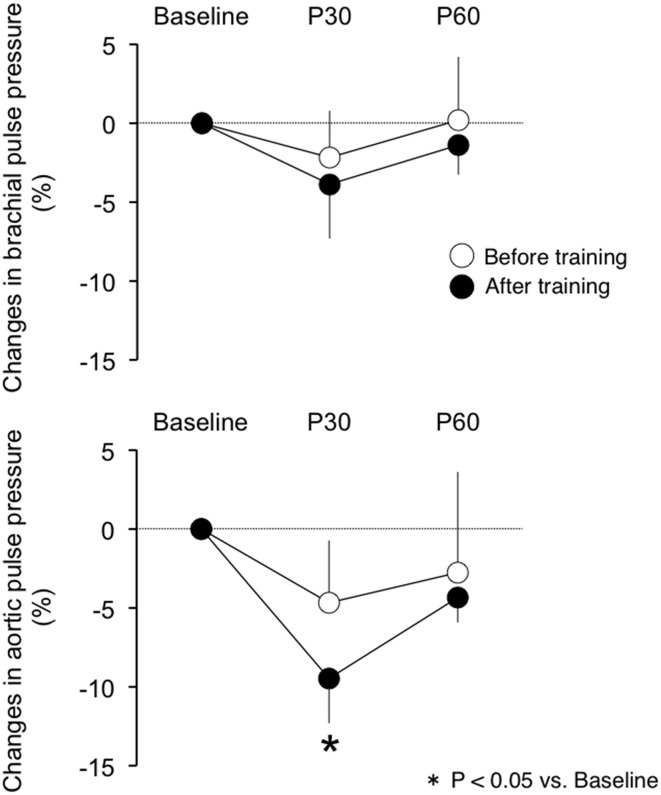
**The post-exercise response of (top) brachial pulse pressure and (bottom) aortic pulse pressure before and after exercise training**. P30, post 30 min acute exercise; P60, post 60 min acute exercise.

**Figure 2:**
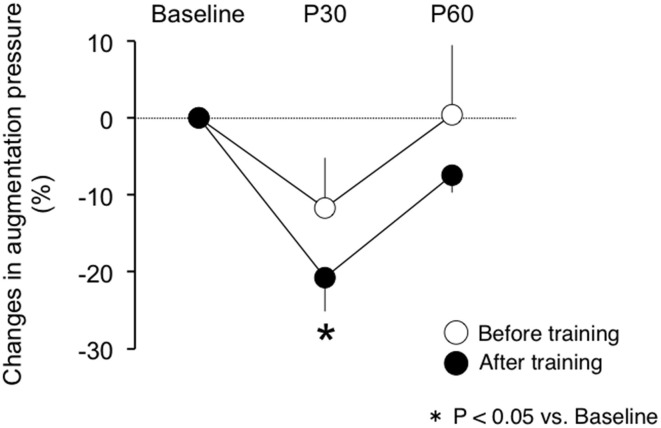
**The post-exercise response of aortic augmentation pressure before and after exercise training**. P30, post 30 min acute exercise; P60: post 60 min acute exercise.

There were no differences in baseline measures of brachial and aortic blood pressure, HR and cfPWV between before and after exercise training (Table 3). Participants completed a 30-min of aerobic cycling exercise test at the intensity of their specific VT before and after the training regimen. Measures of brachial and aortic blood pressure, HR, and cfPWV did not change following the single exercise bout before the training regimen. By use of relative change from the baseline (i.e., pre-exercise), aortic pulse pressures did not change significantly after the initial acute exercise bout, whereas following the training regimen aortic pulse pressures siginificantly decreased with the acute aerobic exercise bout (*P* < 0.05 vs. baseline; Figure 1). The relative changes in brachial pulse pressure with acute exercise bout did not alter significantly either before or after the training. Likewise, the single exercise bout augmentation pressure was not changed before the training regimen but was significantly reduced following the regimen (Figure 2). Significant reductions of these measurements did not last for 60 min.

**Table 3:** **Hemodynamics responses to a single bout exercise before and after training**.

		**Baseline**	**P30**	**P60**
**BRACHIAL**				
Systolic blood pressure (mmHg)	Before	116±4	115±3	115±3
	After	115±3	114±4	116±3
Diastolic blood pressure (mmHg)	Before	71±3	71±2	71±2
	After	69±3	70±3	71±3
Pulse pressure (mmHg)	Before	45±3	43±2	44±2
	After	46±2	44±3	45±3
**AORTIC**				
Systolic blood pressure (mmHg)	Before	108±5	106±3	106±2
	After	106±3	103±4	105±3
Diastolic blood pressure (mmHg)	Before	71±3	71±2	71±2
	After	70±2	70±3	71±3
Pulse pressure (mmHg)	Before	37±4	34±3	35±3
	After	36±3	33±3	35±3
Augmentation pressure (mmHg)	Before	16±2	14±2	15±2
	After	17±2	13±2	15±2
P1_height (mmHg)	Before	21±2	21±1	20±2
	After	20±1	20±1	19±1
Heart rate (beats/min)	Before	61±2	63±2	62±2
	After	58±2	63±2	62±2
Carotid-femoral PWV (cm/sec)	Before	949±21	964±29	970±29
	After	950±30	966±28	987±24

*Data are means ± SE. P1, first systolic peak; PWV, Pulse wave velocity; P30, post 30 min; P60, post 60 min*.

**Figure 3:**
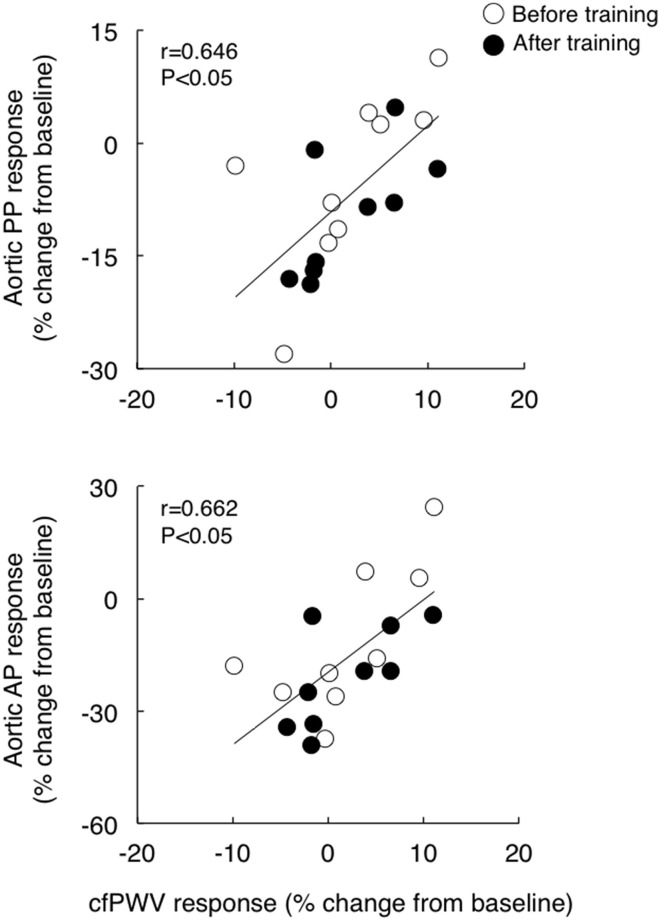
**The relations between the post-exercise response (relative change from the baseline to post 30 min) of carotid-femoral pulse wave velocity (cfPWV) and corresponding aortic pulse pressure (PP, top) and augmentation pressure (AP, bottom) response**.

**Figure 4:**
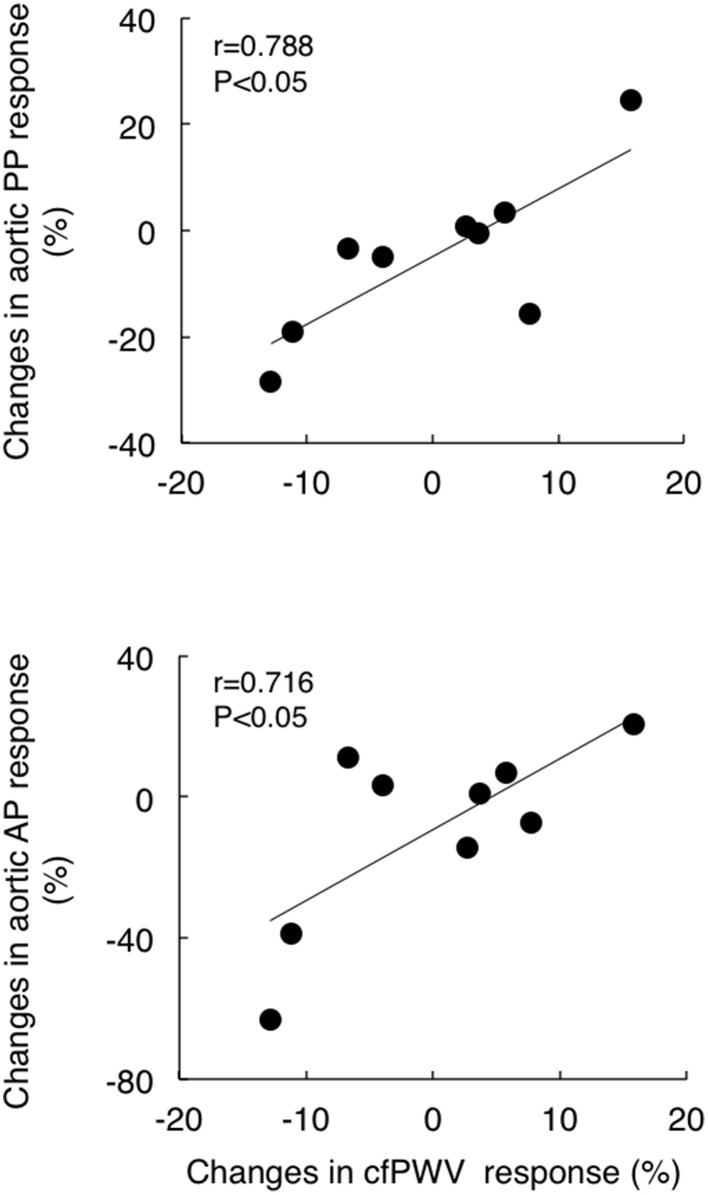
**The relations between training-induced change (from pre- to post-training) in post-exercise response (from the baseline to post 30 min) of carotid-femoral pulse wave velocity (cfPWV) and corresponding changes in aortic pulse pressure (PP, top) and augmentation pressure (AP, bottom) response**.

Although cfPWV did not change significantly with acute exercise bout, the individual post-exercise cfPWV response (post 30 min) correlated with corresponding aortic pulse pressure and augmentation pressure responses (*r* = 0.646 and *r* = 0.662, respectively, Figure 3). Additionally, although baseline cfPWV did not change significantly following the exercise training regimen, the training-induced changes in post-exercise cfPWV responses correlated to the corresponding changes in the aortic pulse pressure and augmentation pressure response (*r* = 0.788 and *r* = 0.716, respectively, Figure 4).

## Discussion

The present study aimed to determine the effect of aerobic exercise training on the acute central hemodynamic response to the single bout of aerobic exercise in postmenopausal women. The main findings are as follows: First, before the training intervention, aortic and brachial BP did not change significantly following 30 min of cycling at the individual VT measured before the training regimen. Second, after the intervention of 12-week exercise training regimen, aortic pulse pressure and augmentation pressure decreased following 30 min of cycling at the individual VT measured after the training regimen. These findings suggest that the central hemodynamic responses to acute aerobic exercise are enhanced by regular aerobic exercise training.

A transient reduction in brachial BP after acute aerobic exercise is reportedly observed in normotensive and hypertensive individuals (Halliwill, 2001; MacDonald, 2002). However, little is known about the central BP response to acute aerobic exercise. We recently reported a study that showed that the aortic pulse pressure decreased with moderate intensity exercise in a group of young healthy men (Sugawara et al., 2015). In the present study, we identified that postmenopausal women exhibited a reduction of aortic pulse pressure following a single bout of aerobic exercise without lowering brachial pulse pressure only after a 12-week training regimen. The present study develops this notion for the aging population. In the present study, the exercise work rate, oxygen consumption, and heart rate during a single bout exercise was increased after exercise training regimen. But, the increases in exercise intensity were not correlated with the central blood pressure response to acute exercise. Thus, it seems that the decrease in aortic blood pressure following acute exercise may be contributed to other than increase in exercise work volume and/or intensity.

In the present study, although there were no significant effects of acute exercise and regular exercise training on cfPWV, temporary cfPWV response to acute exercise is correlated with corresponding response of aortic pulse pressure and augmentation pressure to acute exercise before and after training (Figure 3). Furthermore, the training-induced change in cfPWV response to acute exercise bout was correlated with the corresponding changes in response of aortic pulse pressure and augmentation pressure to acute exercise (Figure 4). These results suggest the changes in arterial stiffness may play a role partially in post-exercise response of central aortic hemodynamics in postmenopausal women.

The underlying mechanism for reduction in aortic pulse pressure may be different between young and older populations. Theoretically, it is believed that the central arterial pressure waveform consists of a forward traveling wave and a later-arriving reflected wave. In young men, the post exercise attenuation of aortic pulse pressure was associated with the corresponding changes in aortic pulse wave velocity (Sugawara et al., 2015), suggesting the delay of the reflected wave mitigates its overlap on the incident wave and lowers aortic pulse pressure. Comparatively, in postmenopausal women, aortic pulse wave velocity did not change after the single aerobic exercise bout either before or after the 12-week exercise training regimen. Additionally, the amplitude of P1 (a surrogate measure of the incident wave amplitude) did not change, whereas augmentation pressure was diminished by the single exercise bout after the training regimen. Conclusively, the significant acute exercise-induced reduction of aortic pulse pressure may be attributed to the attenuated wave reflection rather than delayed return of the reflected wave.

We could speculate several possibilities regarding the mechanism responsible for decreased augmentation pressure in postmenopausal women. Regular endurance training improves endothelial function and capacity of dilation in trained vascular beds in the aging individual (Taddei et al., 2000). Based on the traditional concept that central arterial pressure is comprised of the incident wave (generated by left ventricle) and the reflected wave (emanated from points of impedance mismatch in the lower body) (Nichols and O'Rourke, 2005), the improved vasodilatory function in legs could buffer the incident wave effectively and attenuate the reflected wave. Alternatively, recent studies propose that aortic pressure is affected by, not only the aforementioned components, but also by the reservoir pressure (Wang et al., 2003; Davies et al., 2010). The arterial reservoir pressure is associated with the volume of blood stored in the aorta; it depends on the buffering capacity of the aorta, especially the proximal region (i.e., the ascending aorta and aortic arch). If this emerging idea is true, the ameliorated reservoir function of proximal aorta might be associated with the diminished aortic pressure augmentation. In this study, although cfPWV was not improved by the exercise training, cfPWV mainly reflects pulse wave velocity along the descending aorta and does not cover the ascending aorta and aortic arch.

In the present study, exercise training was found to slightly decrease brachial and central BP at baseline; however, an overall decrease in BP did not reach statistical significance. A meta-analysis had reported that exercise training decreases brachial systolic and diastolic BP by about 3-4 mmHg and 2-3 mmHg, respectively (Kelley and Sharpe Kelley, 2001). On the other hand, the effects of exercise training on central BP are not yet consistent. Laskey et al. (2013) reported that a 20-week exercise rehabilitation program decreased central aortic systolic pressure and pulse pressure at rest in patients with coronary heart disease. However, we and other investigators have shown that aerobic exercise training did not change central aortic hemodynamics in middle-aged and older populations (Tanaka et al., 2002; Sugawara et al., 2006, 2012). These inconsistent results may, in part, be due to the differences in exercise training protocols or subject characteristics among studies. Further studies are needed to clarify the effects of exercise training on central arterial BP at rest.

A number of studies have showed that aerobic exercise training decreases arterial stiffness (Montero et al., 2014). On the other hand, in the present study, we cannot observe the significant change in pulse wave velocity after 12-week training. It should be noted that several studies demonstrated that exercise training did not affect cfPWV in healthy postmenopausal women (Seals et al., 2001; Sugawara et al., 2012). It is likely that elderly women show less response of training effects. Interestingly, Hayashi et al. (2008) suggested that habitual exercise activity decreases arterial stiffness in individual with TC or CC genotype of estrogen receptor-α, there is no significant effect of exercise on arterial stiffness in individual with TT genotype. These results suggest that training efficiency on arterial stiffness might be affected gene polymorphism. However, we could not clarify the precise mechanisms because this was not mechanistic study. The lack of the control group was also a limitation of the study. Randomized controlled trial study is warranted in future study.

In conclusion, we demonstrated that a single 30-min bout of moderate-intensity cycling did not induce a significant change in central aortic pressure in postmenopausal women; but, after a 12-week period of consistent aerobic training, a cycling exercise at relatively similar intensity evoked a significant reduction of aortic augmentation pressure and pulse pressure. These findings suggest that regular aerobic exercise training may improve the blunted central hemodynamic response to an acute aerobic exercise bout in postmenopausal women.

## Author contributions

NA drafted the manuscript. All authors contributed to the interpretation and discussion of the results. NA, SR, and JS contributed data acquisition and analysis. JS and SM provided the concept and design of the study and critically revised the manuscript.

### Conflict of interest statement

The authors declare that the research was conducted in the absence of any commercial or financial relationships that could be construed as a potential conflict of interest.
